# Mental Distress during the COVID-19 Pandemic: A Cross-Sectional Study of Women Receiving the Comprehensive Social Security Allowance in Hong Kong

**DOI:** 10.3390/ijerph191610279

**Published:** 2022-08-18

**Authors:** Jialiang Cui, Vanessa Hoi Mei Cheung, Wenjie Huang, Wan Sang Kan

**Affiliations:** 1 Department of Social Work, The Chinese University of Hong Kong, Hong Kong; 2 Felizberta Lo Padilla Tong School of Social Sciences, Caritas Institute of Higher Education, Hong Kong; 3 Society for Community Organization, Hong Kong; 4 Department of Social and Behavioural Sciences, City University of Hong Kong, Hong Kong

**Keywords:** mental health, COVID-19 pandemic, welfare recipients, Hong Kong

## Abstract

Welfare recipients were often considered the least deserving of COVID-related support. Despite the recent attention paid to the impact of COVID-19 pandemic on mental health, few studies have explored the mental distress experienced by welfare recipients. This cross-sectional study on female Comprehensive Social Security Allowance recipients in Hong Kong aimed to explore their level of mental distress and its association with a range of risk factors specific to welfare recipients. Hence, 316 valid cases from a local community center responded to our online survey. We found that 52.3%, 23.4%, and 78% of the participants showed moderate to extremely severe depression, anxiety, and stress symptoms, respectively. A higher level of mental distress was associated with having a psychiatric diagnosis, poorer social, and greater concerns over disciplining children, the living environment, daily expenses and being infected by COVID-19. Unexpectedly, being married, having a permanent residence, and having a job were not significant protective factors for this group. The models explained 45.5%, 44.6%, and 52.5% of the overall variance in the level of depression, anxiety, and stress (*p* < 0.01), respectively. Our findings have important implications for supporting female welfare recipients during a public health crisis and may help frontline staff and professionals provide prompt assistance to this group in need.

## 1. Introduction

Declared a pandemic by the World Health Organization in 2020, COVID-19 has had an enormous global impact on people’s lives. As of June 2022, the cumulative number of COVID-related deaths was over 6 million worldwide. In Hong Kong, the six waves of the pandemic have led to over 1.2 million confirmed cases and nearly 10,000 deaths [1]. The rapid and continuing transmission of the virus has also resulted in severe economic and social disruptions. Stringent social-distancing measures and travel restrictions were imposed, perpetuating social problems, such as food insecurity, family separation, social isolation, and school closures [2].

Unsurprisingly, an increasing prevalence of mental distress has accompanied these economic and social outcomes. During the initial phase of the pandemic, a systematic review by Vindegaard and Benros [3] concluded that the public’s psychological well-being had decreased significantly from its pre-pandemic level. Hong Kong’s situation reflected the international situation. Mahmud et al. [4] conducted a meta-analysis in this area and found that the global prevalence of depression, anxiety, stress increased significantly (28.18%, 29.57% and 25.18%, respectively). Hong Kong is no exception. According to Zhao et al. [5], symptoms of stress increased significantly among the people of Hong Kong by 28.3% after the outbreak, prevalence of anxiety increased by 42.3%, and the largest increase was found for depression, which was twice as high as in 2016–2017. Without timely intervention, these mental health issues can lead to more devastating consequences (e.g., suicide) and continue to affect people’s lives even after the pandemic.

Accordingly, there has been enormous focus on investigating the factors that contributed to deteriorating mental health during the pandemic across a wide range of target groups. The literature has repeatedly emphasized the factor of financial difficulties in cross-national settings and diverse target group samples. Worsening finances, along with their associated causes and outcomes, such as unemployment, lower income, lower savings, and housing insecurity, led to a higher risk of mental distress [6,7,8,9,10,11], highlighting the importance of providing mental health initiatives and financial interventions together. Accordingly, measures to enhance the social security system’s response to COVID became a major strategy to alleviate the pandemic’s financial difficulties and their impacts. In 2021, for example, Hong Kong’s government temporarily relaxed the eligibility criteria for Comprehensive Social Security Assistance (CSSA), a temporary program for people in financial hardship, for able-bodied persons and issued an extra one-time grant to CSSA recipients. Although these measures might have eased the financial burden on citizens in need, at least to an extent, they also directed attention away from welfare recipients, obscuring their needs in other domains.

Welfare recipients’ mental health needs during the pandemic have been largely omitted from the current literature. The literature exploring mental distress and its risk factors covers not only the general population but also a wide range of groups such as the elderly [12], pregnant women [13], young adults [14], homeless people [15], health workers [16], university students [8], low-income groups and immigrants [10]. To our knowledge, however, no mental health studies have focused on welfare recipients’ needs during the pandemic, perpetuating the public’s indifferent attitude toward this group [17,18] at least to a degree. Blanchet and Landry [19] compared the public’s attitudes toward welfare recipients before and during the pandemic and found that welfare recipients were deemed the least deserving of additional support at both time points. Consequently, welfare recipients were frequently excluded from support programs that responded to the impact of COVID [20].

To fill the literature gap, this study explores how the mental health of welfare recipients was affected during the pandemic and the associated risk factors. This investigation has important relevance to the Hong Kong context. In Hong Kong, the CSSA offers financial protection to citizens experiencing unemployment, low earnings, disability, ill health, single parenting and old age [21]. However, the program has always been touted as a last resort, not only by the government (which has maintained a small-government policy for decades), but also by the public due to its widespread prejudice against CSSA recipients. Mainstream media and policy documents often associate Hong Kong citizens with the stereotype of tenacious, self-reliant “Hongkongers” rendering a negative image for CSSA recipients, particularly able-bodied persons, as “lazy” and “irresponsible” [22]. Recent report shows that many unemployed people affected by the pandemic were unwilling to apply for welfare payments due to their fear of welfare stigma [23]. As a result, many citizens only apply for CSSA after long-term unemployment or when they have complex health and social problems. During COVID, CSSA recipients were more likely to struggle with both increased living costs and their various disadvantages, both of which were magnified by the impact of the pandemic. Unfortunately, their cry for help was easily drowned out by Hong Kong’s deeply seated stereotype of “greedy” welfare recipients.

### Potential Factors Associated with the Mental Distress of Welfare Recipients during the Pandemic

In this study, the notion of “mental distress” was used to refer to psychological conditions such as anxiety, depression and stress, ranging from mild to severe levels. These are the three most commonly documented conditions in the literature on the experience of mental distress during the pandemic [24,25]. The existing literature, particularly the recent meta-analyses by Balakrishnan et al. [26], Metin et al. [27], and Bello et al. [28], has proposed that various risk factors contribute to the prevalence of mental distress across distinctive groups, including sex, history of existing medical conditions, age, culture, fear of infection, employment status, social support, etc. Among these risk factors, a critical predictor to worsening mental distress during the pandemic, as shown in many studies [3,7,11,28], was suggested to a pre-existing mental illness. This may be a critical variable given that a large proportion of welfare recipients meet the clinical criteria for mental illness [29].

Furthermore, consistent evidence has demonstrated that females were more vulnerable than males to mental distress during the pandemic [6,7,10,30]. Accordingly, female CSSA recipients were identified as our key target group. Because of Hong Kong’s inadequate childcare support and improper service delivery, younger mothers’ participation in the workforce decreased from 2007 to 2018 [31]. Women are often the family caregivers, a responsibility that prompts many to give up their full-time jobs. The tremendous challenges of caregiving caused by COVID-related disruptions (e.g., school closures) warrant an investigation that focuses on these female recipients. Furthermore, being married and having children to care for may also affect their perceived caregiving pressure.

Employment status has also been found to be a significant variable associated with mental distress in different countries, with employment being a protective factor against stressful situations [6,9]. This protective effect requires further exploration in the context of women on CSSA, who often act as informal caregivers and sometimes work in part-time or casual positions in the labor market.

Social support has been suggested as another consistent predictor of mental health [6,7,14]. This concept refers to individuals’ perception of the coping assistance available to them from members of their social network, such as family, friends and significant others (e.g., colleagues) [32]. According to the stress-buffering model, social support is a crucial factor that can buffer the impact of stressful events on a person’s mental health [33]. However, the social networks of women on CSSA in Hong Kong might be weak because they are so stigmatized. CSSA recipients perceive that people in Hong Kong show little sympathy for their hardship [22], indicating that their inner familial circles are their main source of support. Furthermore, over 10% of CSSA recipients are new immigrants [22], a group that lacks social support regardless of stress level, gender and income [34]. Thus, we are reminded to take residency status into consideration.

Housing insecurity might have been a significant risk factor for mental distress during the pandemic [10]. In Hong Kong, recent research showed that 84.2% of the deterioration in people’s mental health can be attributed to housing affordability alone [35]. Although some CSSA recipients receive a rental allowance, the extremely small living space (e.g., 51 ft per person in subdivided flats) [36] of subsidized housing intensified existing conflicts and pressure, especially during the lockdown period.

The literature has also suggested a range of pandemic-related stressors that put people at greater risk of mental distress. The most commonly highlighted stressor is concern about becoming infected [2,6,30,37], which predicts worse mental distress in both the Western and the Eastern contexts. In addition to this fear of COVID-19, subpopulations with distinctive needs may suffer from different pandemic-related stressors. For example, a study of university students assessed 13 stressors associated with the pandemic, including COVID infection, living expenses, social interactions and campus life [8]. However, pandemic-related stressors are often constructed based on literature reviews. In this study, we assessed stressors based on both the literature and a discussion with a local grassroots NGO that serves community residents, including CSSA recipients.

In sum, the purposes of this study were (1) to assess the level of mental distress experienced by female CSSA recipients, a group that has been excluded from previous pandemic research, and (2) to identify the risk factors associated with these women’s mental distress. Analyses of this study were exploratory, and the data collected were used to examine the mental health outcomes of welfare recipients and illustrate the relationship between these outcomes and demographics, living environment, social support and pandemic-related stressors. Findings are expected to contribute to informing the design of specific, contextualized welfare interventions during a public health crisis.

## 2. Method

### 2.1. Participants

A cross-sectional study was conducted among female CSSA recipients to address our research aims. We administered an online survey using Qualtrics from April to August 2021. Because of the stigmatization of welfare recipients in Hong Kong, many people choose to conceal their status as CSSA beneficiaries [22]. Given this potential recruitment difficulty, our primary recruitment strategy involved close collaboration with a local grassroots NGO, the Society for Community Organization (SoCO), which is the largest organization in Hong Kong serving economically and politically disadvantaged groups. Advertisements for the study were sent through SoCO’s network, and participants who encountered technical issues were helped to complete the survey by SoCO’s staff. Overall, 405 female CSSA recipients accessed the online survey; 316 completed the essential questions about demographic information, mental health measures, social support conditions and pandemic-related stressors and thus were included in our final dataset for this analysis. The study was approved by the Social Behavioral Research Ethics panel of the Chinese University of Hong Kong (SBRE-20-589).

### 2.2. Measures

***Demographics***. All of the participants were female. The demographic information was dummy coded into variables for marital status (1 = married; 0 = other), whether the participants had children under the age of 18 (1 = yes; 0 = no), residency status (1 = permanent resident; 0 = nonpermanent resident) and employment status during the pandemic (1 = currently employed currently; 0 = not currently employed).

***Living environment***. The participants were asked to indicate where they lived during the pandemic. Reflecting the diverse housing environments in Hong Kong, this item was measured on the following seven categories: public housing (1), subdivided flat with separate bathroom (2), cubicle or cage home with shared bathroom (3), friends’ or relatives’ home (4), self-owned property (5), social housing (6), and other (7).

***Mental distress***. First, we assessed the severity of the participants’ depression, anxiety and stress symptoms using the Chinese version of the 21-item Depression Anxiety Stress Scale (DASS-21) [38], which has been widely used in the Hong Kong context [24,39]. The scale was structured into the following three subscales: depression, anxiety and stress. Each item was rated on a 4-point Likert scale, ranging from “did not apply to me at all” to “applied to me very much or most of the time” to measure the condition of each participant’s mental state over the previous week. The scores for the overall DASS-21 and each of its subscales were calculated and then categorized into the corresponding 5 severity labels from mild to extremely severe. The Cronbach’s *α* of the DASS-21 and its three subscales in this sample were 0.959 (total), 0.893 (depression), 0.889 (anxiety) and 0.897 (stress). Second, the participants were asked if their mental distress was different from how it had been before the pandemic (1 = worse than before; 2 = the same; 3 = better than before). In addition, the participants’ history of psychiatric diagnosis was explored with a dummy variable (0 = no diagnosis of mental illness; 1 = have been diagnosed as having mental illness).

***Social Support***. The Chinese version of the Multidimensional Scale of Perceived Social Support (MSPSS) was adopted in this survey [40]. Each scale item was assessed on a 7-point Likert scale ranging from “very strongly disagree” to “very strongly agree.” The MSPSS comprises three subscales that assess the support that participants receive from family, friends and significant others [41]. The total scores for social support and support on each dimension are shown by the item means for the entire scale and each subscale. The Cronbach’s α of the MSPSS and its subscales in this sample was 0.914 (total), 0.832 (significant others), 0.828 (family) and 0.847 (friends).

***Pandemic-Related stressors***. We explored a list of pandemic-related stressors based on a literature review and the research team members’ work experience. This list included the stressors highlighted by the previous research related to COVID infection, daily expenses, purchasing anti-pandemic supplies and the living environment. Additional stressors that reflected the target group’s potential concerns and the local context included worries about interacting with spouses, interacting with senior family members, supervising children’s studies, disciplining children and returning to mainland China. The participants were also asked if they had experienced other stressors during the pandemic, but all of the answers were found to repeat the above nine stressors. All of the items were measured on a 4-point Likert scale from “not worried at all” (0) to “extremely worried” (3). The Cronbach’s *α* of the entire scale was 0.813.

### 2.3. Data Analysis

Descriptive statistics were obtained to summarize the participants’ sociodemographic information and the distributions of the variables of interest. The most common stressors for the female CSSA recipients during the pandemic were highlighted. Second, bivariate correlations were examined between the study variables to identify high correlations between the predictors, which can lead to severe multicollinearity. To enable parallel consideration of nominal, ordinal, and interval variables, we employed ϕK coefficient, a practical correlation coefficient based on refinements to Pearson’s χ^2^ contingency test of independence of two variables [42]. ϕK coefficient, ranging from 0 to 1, can indicate the strength of both linear and non-linear correlations. For bivariate normal distributions, it is very similar to the strength of Pearson’s correlation coefficient [42], although it does not indicate direction. Due to the lack of a gold standard of correlation threshold for the ϕK coefficient, we defined scores greater than 0.7 to be high correlation, drawing on the standard of Pearson’s correlation coefficient [43]. Statistical significance was evaluated based on a modified *p*-value algorithm which calculates a Z-value for each cell of the correlation matrix [44]. Z-value corresponding to the threshold *p*-value of 0.05 was used to determine significance in this paper [42].

Finally, a multiple linear regression analysis was performed in three steps to examine the relationships between various risk factors and mental distress. The model was first controlled for marital status, residency status and whether the participants had children (step 1). Additional literature-informed risk factors were added, including social support, type of living environment, psychiatric diagnosis and household employment status (step 2). Common pandemic-related stressors were also added (step 3). The total scores of the three DASS-21 subscales were treated as continuous variables to generate a more precise prediction. Nominal variables have been recoded into dummy variables [45]. The issue of multicollinearity was also investigated with the variance inflation factor (VIF) and the condition index. ϕK coefficient was calculated using the PhiK (ϕK) package with python, and other analyses were performed using the software IBM SPSS Statistics 26, with the statistical significance set at 0.05.

## 3. Results

Of the 316 female CSSA recipients in the sample, 34.5% were married, and 34.8% were not permanent Hong Kong residents (their length of stay in Hong Kong was less than 7 years). Only 13.6% of the women were employed (including part-time and casual work) during the pandemic. Moreover, 41.5% of participants lived in public housing, the regular public housing program for low-income families in Hong Kong, while the remainder lived in other conditions with the majority in subdivided flats. This variable of housing type was recoded into a dichotomous variable (1 = public housing; 0 = other) for our subsequent analysis. The majority of the participants (96.2%) took care of at least one child under the age of 18. With respect to mental illness, 40.8% of the participants had been previously diagnosed. The average item mean of the MSPSS was 3.92 (SD = 1.13), with the highest-rated support coming from significant others (Mean = 4.04; SD = 1.26). Detailed information is shown in Table 1.

With respect to the severity of mental distress, 56.6% of the participants reported that their mental health was worse than it was before the pandemic, and 37.5% reported that it was the same. The mean scores of the DASS-21 were 22.6 (SD = 13.7; for total subscale), 6.9 (SD = 4.9; for depression), 6.6 (SD = 4.6; for anxiety) and 9.1 (SD = 4.8 for stress). Notably, as shown in Table 2, 65.5% of the participants exhibited elevated symptoms of depression (i.e., from mild to extremely severe), 37% showed elevated symptoms of anxiety and 56.4% reported severe or extremely severe symptoms of stress, based on the cutoff score of the DASS-21 [38].

The common pandemic-related stressors rated by the participants as creating a moderate level of concern (mean > 1.5) were (1) supervising children’s studies, disciplining children, the inability to return to mainland China, being infected, and the living environment. Items with a mean lower than 1.5 were not added to the regression analysis. Details are provided in Table 3.

### Regression Analysis

Table 4 shows the correlation between all of the risk factors and the total DASS-21 score using ϕK coefficient. High correlations were found in the relationship between the items of concern about supervising children’s study and disciplining children, along with the relationship among the three dimensions of social support. Therefore, the total score of the MSPSS (instead of each subscale score) and the mean score for concern about disciplining children (which had a stronger correlation with the DASS score) were included in the regression analysis. The VIF statistics showed appropriate tolerance levels for the variables included in the model. The condition index of the model (approximately 24 for all three of the outcome variables) was under 30.

The results of the hierarchical regression analyses are shown in Table 5. In the main effect models (step 3), having a psychiatric diagnosis (*p* < 0.001), increased concerns about daily expenses (*p* < 0.01), the living environment (*p* < 0.01), disciplining children (*p* < 0.001) and being infected (*p* < 0.01) were associated with higher levels of every aspect of mental distress. Having stronger social support may have alleviated the symptoms of depression (*p* < 0.01) and stress (*p* < 0.05). The final reduced models explained 46.2% of the overall variance in depression (*p* < 0.001), 45.6% of the overall variance in anxiety (*p* < 0.001), and 52.7% of the overall variance in stress (*p* < 0.001).

## 4. Discussion

Drawing on a sample of female CSSA recipients, this study assessed the mental distress of welfare recipients, who have been a largely overlooked group during the pandemic. First, the findings paint an alarming picture for Hong Kong compared with studies using the DASS-21 scale to assess mental distress during the pandemic in regions such as mainland China, Spain, Turkey, and Bangladesh (depression: 3.7–43%; anxiety: 3.9–26%; stress: 1.5–32%) [7,30,46,47]. In Hong Kong, a study by Tso and Park [39] on the psychological distress of the general population found that 50.4%, 28.1% and 59.6% of the participants showed moderate to extremely severe depression, anxiety and stress, respectively. Although female welfare recipients reported levels of depression (52.3%) and anxiety (23.4%) similar to those of the general population, they experienced a higher level of stress (78%) than the general population. The main difference between the participants and the general population lies in the group of people who reported an extremely high level of stress, with 25% falling into this group in the sample of the general population and 45.6% falling into this group among the participants of this study.

Exploring what might have contributed to this high level of mental distress among the participants, we found that having a previous psychiatric diagnosis was one of the most significant risk factors for all three aspects of mental distress. This finding is consistent with previous studies’ findings that the presence of psychiatric illnesses predicts worse mental health [3,7,11]. A recent study in Hong Kong suggested that 24.2%, 32.6%, and 18.9% of psychiatric patients presented with severe or extremely severe depression, anxiety and stress symptoms, respectively, during the pandemic [48]. Female welfare recipients seem to be more vulnerable to developing symptoms of stress, which is shown in the regression analysis as relatively less predictable when a participant has a psychiatric diagnosis compared to the other two forms of distress.

Furthermore, the study shows that the prevalence of mental illness diagnosis (40.8%) among female welfare recipients is much higher than the estimated prevalence of common mental disorders in Hong Kong (13.3%) according to a population-based survey [49]. Although this number may seem high, it is similar to estimates regarding the mental health outcomes of welfare recipients generated in other studies. For example, in Australia, Butterworth [50] found that one in three income support recipients had a diagnosable mental illness, and the prevalence among single recipients with children was even higher (45%). Future studies in Hong Kong should continue to monitor the mental health outcomes of welfare recipients.

Another significant predictor of mental distress during the pandemic was the level of social support perceived by the participants. Our findings suggest that greater social support may help address depressive and stress symptoms, as found both in the study of Liu et al. [37] on a sample of the Chinese general population and in studies conducted in other countries [6,7,14]. However, although social support has been found to protect against anxiety during the pandemic [51,52], no significant effect was identified in this study. This may be due to the mixed effect of social support. As some research has suggested, the receipt of a substantial amount of social support can lead to self-doubt [53], which in turn contributes to increased rates of anxiety [54]. In addition, the participants with more social support had to worry about more family members or friends than did the participants with less social support.

It was also found that the participants’ support networks were relatively poor, with a total mean MPSS score of 3.92, much lower than the 4.75 that has been found for new immigrants [55] and the 5.33 for university students [56] in Hong Kong, not to mention the 5.3–5.5 for the Chinese general population during the pandemic [57]. Unexpectedly, residential status was not related to all of the dimensions of social support, indicating that poor social support is a universal problem for female welfare residents regardless of whether they are new immigrants. The stereotyping of and indifferent attitudes toward welfare recipients in Hong Kong may have dramatically undermined the participants’ opportunities to be helped and motivation to seek support. Nonetheless, exploring the results of each dimension of social support, we found that contrary to our expectations, the strongest support reported by participants came from significant others, with family support rating as the least helpful. This can be explained by the large proportion of unmarried participants in this sample. Participants’ connection with the local service agency which helped in recruitment may also, to some degree, explained that significant others (e.g., agency staff and other women on welfare) were rated the strongest support. The finding also indicates the importance of active service engagement and peer support to improving the quality of social support for female welfare recipients.

A range of pandemic-related stressors explains the changes in mental distress in our analysis. Most previous studies have focused on the prediction of the number of stressors that create mental distress [8,24], whereas our analysis explored each of those stressors in detail. The most significant of these stressors for all three aspects of mental distress was concern about disciplining children. The Hong Kong government repeatedly suspended and reduced in-person classes, school activities and children care services (e.g., after school care program and child/youth centers) in response to the outbreak. Parents had to spend more time and effort disciplining their children, who stayed at home more than they did before. Our participants, with most being single mothers, may experience greater hardship in child rearing, as they tend to have limited finance and familial resources. As shown in the regression analysis, this concern over disciplining children constitutes the most significant factor that exacerbates the stress level of these women. Moreover, because welfare recipients tend to have fewer digital resources, they may also have worried about their children’s ability to adapt to online learning and to keep up with the learning process. This is demonstrated by the rating of their concern about their children’s studies as the most important stressor.

In addition, concerns about being infected, daily expenses and the living environment were found to increase all forms of mental distress. These findings are in line with previous research that highlighted the impact of worrying about infection and financial difficulties [2,6,30,37]. Several important findings should be noted here. First, although the participants’ concern about their living environment predicted their mental distress, where they live (i.e., housing type) was not significantly related to that distress, indicating the importance of subjective adaptivity to one’s housing environment over the impact of the physical environment. Second, for female welfare recipients, concern about financial difficulties was the most significantly associated with concern about caregiving (*p* < 0.01). The cost of online learning, including the purchase of Internet data, computers, tablets, and other learning materials, may have dramatically increased their daily expenses.

Although previous research has often highlighted the protective effect of employment [6,9], we found that employment had no effect on our participants’ mental distress, possibly because most of the participants were unmarried (65.6%) but had to take care of at least one child (96.2%). With their children at home during the pandemic, these mothers may have chosen to live mainly on CSSA instead of engaging in employment. Even if they had intended to work, their likelihood of finding employment would have been low. Hong Kong’s labor market is under tremendous pressure, with a seasonally adjusted unemployment rate of 7.2% from December 2020 to February 2021, the highest since 2004 [58]. The most impacted industries include services, restaurants and retail, which usually offer many casual or part-time jobs with lower requirements of qualifications.

Some limitations of this study should be noted. Because of the difficulty in recruiting welfare recipients and limited resources, random sampling was not conducted, and the sample size was relatively moderate. Our use of the nonprobability sampling method might also have undermined the external validity of this study. Secondly, the scale of pandemic-related stressors was constructed based on literature and expert opinion. While its reliability turned out to be good, the scale was not validated in any research of pilot testing. Thirdly, this study explored the mental health status of participants in the middle of 2021, when the Hong Kong government was gradually relaxing strict social-distancing measures; conditions during the lockdown period might have been worse. These limitations highlight the need for a longitudinal study of this issue. Our use of a cross-sectional design is not sufficient to confirm the direction of the causal relationships between the variables. Finally, because our study did not offer reimbursement to the participants, the questionnaire needed to be relatively short, and some variables were therefore not included (e.g., education, age, mental health literacy and caregiving capacity). We suggest that future research continue to explore the factors that may lead to improved mental health outcomes for this marginalized group after the pandemic.

Despite these limitations, this study is, to our knowledge, the first to explore the issue of the mental distress of female welfare recipients during the pandemic. Our findings have important implications for how to support female welfare recipients during a public health crisis. Most of our participants reported elevated symptoms of mental distress, with a much higher level of stress compared to the general population in Hong Kong. It is of great importance for the government to recognize the elevated level of stress among female recipients during a public health crisis, and to create timely measures to address the major source of toxic stress, such as financial burden, caregiving difficulties and a lack of social support. Moreover, it should be noted that 40.8% of the participants reported a pre-existing diagnosis in this survey, reflecting a need for more investment in monitoring mental health outcomes of this group and offering professional mental health support. Social support is also key to improving emotional well-being. Because our findings suggest most female CSSA recipients are single mothers, one promising approach would be to strengthen and broaden these women’s networks of friends and community support. This support should be accessible and flexible to accommodate the busy schedule of these mothers (e.g., organizing tailor-made services or peer activities beyond normal working hours). More importantly, it is clear that the participants’ most significant risk factor was their concern about caregiving, which can also lead to increased concern about daily expenses. These factors’ impact on mental distress cannot be mitigated by employment, and there is therefore a need for interventions to facilitate the learning and discipline of the children in these families. The government should strengthen the services of childcare centres and after-school care to cover more families during the pandemic, particularly grassroot families in struggle. Finally, the standard payment rates under the CSSA were set by the Social Welfare Department in 1996 based on the public’s basic needs. However, the CSSA allowance should cover essential, but extra expenses during a pandemic, such as online learning materials and anti-pandemic supplies.

## 5. Conclusions

This study shows that female welfare recipients in Hong Kong experienced elevated symptoms of depression and anxiety and a higher level of stress compared with most population groups during the COVID-19 pandemic. A large proportion of the participants had a pre-existing diagnosis of mental illness and a poor social support network, putting them at greater risk of more severe mental distress in the context of COVID. Numerous pandemic-related stressors exacerbated this distress, the most significant of which was concern about disciplining children. Our findings suggest that in addition to mental health interventions, broadening social networks, supporting caregiving, and increasing CSSA coverage may protect female welfare recipients against mental distress during a pandemic.

## Figures and Tables

**Table 1:** Demographic Characteristics of Participants and Social support.

Variables	n	%	
Marital status			
Married	109	34.5	
Others	207	65.6	
Residency status			
Permanent	110	34.8	
Not permanent	206	65.2	
Household employment status			
Being employed currently	43	13.6	
Not working currently	273	86.4	
Housing type			
Public housing	131	41.5	
Subdivided flat	158	50.0	
Cubicle or cage home	10	3.2	
Friends’ or relatives’ home	2	0.6	
Self-owned property	8	2.5	
Social housing	1	0.3	
Others	6	1.9	
Whether has children			
No	12	3.8	
Yes	304	96.2	
Having a mental illness diagnosis			
Yes	129	40.8	
No	187	59.2	
		*M*	*SD*
Social support		3.92	1.13
Support from family		3.80	1.31
Support from friend		3.89	1.23
Support from significant others		4.04	1.26

*Note*. N = 316, calculated using the cut-off score for each subscale of DASS-21.

**Table 2:** DASS-21.

Variables	Depression	Anxiety	Stress
	%	%	%
Normal	34.5	63	13.9
Mild	13.3	13.6	7.9
Moderate	32.3	12	21.8
Severe	9.2	7.9	10.8
Extremely severe	10.8	3.5	45.6

*Note.* N = 316, calculated using the cut-off score for each subscale of DASS-21.

**Table 3:** Common Pandemic-related Stressors.

Variables	*M*	*SD*
Supervising children’s study	1.97	0.92
Disciplining children	1.95	0.91
Unable to return to Mainland China	1.91	1
Being infected	1.89	0.89
Daily expenses	1.83	0.77
Living environment	1.51	1.04
Purchase of anti-epidemic supplies	1.29	0.86
Interaction with spouse	0.89	1.06
Interaction with senior family members	0.68	0.86

**Table 4:** Correlation Between Variables.

	1	2	3	4	5	6	7	8	9	10	11	12	13	14	15	16
1. Marital status	1.00	0.00	0.20	0.00	0.22 *	0.09	0.02	0.20 *	0.16	0.00	0.07	0.00	0.00	0.00	0.00	0.06
2. Residency status		1.00	0.12	0.41 *	0.00	0.00	0.00	0.00	0.15	0.00	0.21 *	0.00	0.07	0.00	0.22 *	0.13
3. Whether has children			1.00	0.00	0.00	0.00	0.15	0.16	0.12	0.19	0.18	0.28 *	0.34 *	0.00	0.00	0.18
4. Housing type				1.00	0.06	0.33 *	0.08	0.07	0.13	0.00	0.54 *	0.00	0.00	0.04	0.20	0.00
5. Household employment status					1.00	0.00	0.00	0.00	0.00	0.00	0.00	0.16	0.00	0.14	0.00	0.19
6. Psychiatric diagnosis						1.00	0.23 *	0.00	0.00	0.24 *	0.19 *	0.22 *	0.29 *	0.28 *	0.11	0.49 *
7. Social support (significant others)							1.00	0.78 *	0.78 *	0.11	0.00	0.14	0.10	0.00	0.00	0.40 *
8. Social support (family)								1.00	0.77 *	0.10	0.24 *	0.21 *	0.19	0.28 *	0.00	0.33 *
9. Social support (friends)									1.00	0.00	0.00	0.17	0.18 *	0.17 *	0.02	0.33 *
10. Concerns about daily expenses										1.00	0.42 *	0.50 *	0.48 *	0.55 *	0.50 *	0.53 *
11. Concerns about living environment											1.00	0.34 *	0.31 *	0.50 *	0.47 *	0.34 *
12. Concerns about children’s study												1.00	0.89 *	0.50 *	0.46 *	0.49 *
13. Concerns about disciplining children													1.00	0.50 *	0.43 *	0.53 *
14. Concerns about being infected														1.00	0.56 *	0.41 *
15. Concerns about returning to Mainland China															1.00	0.30 *
16. DASS-21 total score																1.00

* *p* < 0.05.

**Table 5:** Regression Analysis.

Predictors	Depression Standardized Coefficient			Anxiety Standardized Coefficient			Stress Standardized Coefficient		
	Step 1	Step 2	Step 3	Step 1	Step 2	Step 3	Step 1	Step 2	Step 3
1. Marital status	−0.04	0.03	0.04	−0.05	0.02	0.03	−0.04	0.02	0.03
2. Residency status	−0.02	−0.02	−0.03	−0.03	−0.02	−0.03	0.01	0.03	0.01
3. Whether has children	−0.02	−0.02	−0.07	−0.01	−0.01	−0.05	0.04	0.03	−0.04
4. Housing type		0.02	−0.09		0.02	−0.09		0.06	−0.07
5. Household employment status		−0.09	−0.07		−0.09	−0.07		−0.06	−0.03
6. Psychiatric diagnosis		−0.37 #	−0.22 #		−0.42 #	−0.27 #		−0.35 #	−0.19 #
7. Social support		−0.19 #	−0.12 #		−0.12 #	−0.04		−0.19 #	−0.09 *
8. Concerns about daily expenses			0.18 #			0.14 #			0.21 #
9. Concerns about living environment			0.14 #			0.25 #			0.20 #
10. Concerns about disciplining children			0.27 #			0.23 #			0.35 #
11. Concerns about being infected			0.13 #			0.15 #			0.15 #
12. Concerns about returning to Mainland China			0.23			0.06			0.01

# *p* < 0.01, * *p* < 0.05.

## Data Availability

The data presented in this study are available on request from the corresponding author. The data are not publicly available due to privacy restriction.
